# Effect of a processing delay between direct and delayed sound in simulated open fit hearing aids on speech intelligibility in noise

**DOI:** 10.3389/fnins.2023.1257720

**Published:** 2024-01-04

**Authors:** Sebastian Roth, Franz-Ullrich Müller, Julian Angermeier, Werner Hemmert, Stefan Zirn

**Affiliations:** ^1^Department of Electrical Engineering, Medical Engineering and Computer Science, Peter Osypka Institute of Medical Engineering, Offenburg University of Applied Sciences, Offenburg, Germany; ^2^Department of Electrical and Computer Engineering, Munich Institute of Biomedical Engineering, Technical University of Munich, Munich, Germany

**Keywords:** binaural hearing, speech in noise, open fitting, unilateral hearing loss, speech intelligibility model

## Abstract

**Introduction:**

Subjects with mild to moderate hearing loss today often receive hearing aids (HA) with open-fitting (OF). In OF, direct sound reaches the eardrums with minimal damping. Due to the required processing delay in digital HA, the amplified HA sound follows some milliseconds later. This process occurs in both ears symmetrically in bilateral HA provision and is likely to have no or minor detrimental effect on binaural hearing. However, the delayed and amplified sound are only present in one ear in cases of unilateral hearing loss provided with one HA. This processing alters interaural timing differences in the resulting ear signals.

**Methods:**

In the present study, an experiment with normal-hearing subjects to investigate speech intelligibility in noise with direct and delayed sound was performed to mimic unilateral and bilateral HA provision with OF.

**Results:**

The outcomes reveal that these delays affect speech reception thresholds (SRT) in the unilateral OF simulation when presenting speech and noise from different spatial directions. A significant decrease in the median SRT from –18.1 to –14.7 dB SNR is observed when typical HA processing delays are applied. On the other hand, SRT was independent of the delay between direct and delayed sound in the bilateral OF simulation.

**Discussion:**

The significant effect emphasizes the development of rapid processing algorithms for unilateral HA provision.

## 1 Introduction

The common assumption for hearing loss (HL) is often a bilateral HL with a near-symmetric hearing threshold on both sides. Therefore, providing hearing aids (HAs) on both sides is the established procedure of HA provision, specified in the DIN EN ISO 21388 standard (DIN German Institute for Standardization, 2022). However, this assumption is not valid for all HL. A bilateral HL is classified as asymmetric if the HL difference is equal to or larger than 15 dB between the hearing thresholds of both ears (Le et al., 2017). Furthermore, in the latest report, the World Health Organization (WHO) classified a unilateral HL as a new grade of HL. The WHO provides this classification with a hearing threshold larger than 35 dB in one ear with a contralateral normal-hearing ear (World Health Organization, 2021).

The prevalence of unilateral HL is not easy to estimate as there are few publications on this topic. From the dataset of a study by Von Gablenz and Holube (2019), the proportion of people with unilateral HL in the hearing-impaired population can be extracted as 8.2% and in the total population over 49 years of age as 13.3%, referring to the study by Chia et al. (2007). Nevertheless, the treatment of unilateral HL is important. This was shown, e.g., by Hoppe et al. (2022), who demonstrated a detrimental effect of asymmetric HL on word recognition that increases with increasing asymmetry. Further evidence comes from Kurioka et al. (2021), who showed that patients with an asymmetrical HL achieve a worse monaural word discrimination score in the ear with the more severe HL than the monaural word discrimination score in patients with bilateral HL of the same severity. This can be explained by the non-use and resulting deprivation of the worse hearing ear. The authors conclude that more attention should be paid to the treatment of asymmetric HL. Furthermore, an early intervention of HL could be beneficial for treating asymmetric HL (Kurioka et al., 2021; Hoppe et al., 2022).

For a unilateral HL, the treatment is often unilateral HA provision. Compared to the limited data availability of unilateral HL, the number of unilateral HA provisions can be determined more reliably. In the study from, Anovum EuroTrak Germany (2022), 26% of the HA users were unilaterally fitted. This percentage includes all unilateral provisions. However, there can be multiple reasons for such unilateral provisions, among which unilateral HL is one. Other factors can be sequential provision because of the standard procedure for familiarization, cosmetic, cost factors, or the occurrence of binaural interference in speech perception in elderly HA users (Walden and Walden, 2005; Hoppe et al., 2022). Further data from Holube et al. (2019) encountered 42 unilateral HA users in a group of 196 randomly selected HA users. A unilateral HL grade with a near normal-hearing contralateral ear was found in nine subjects. The data resulted in 4.5% of HA users with a WHO-classified unilateral HL among all HA users in the study. In addition, the Anovum EuroTrak study shows a lower adoption rate for unilateral HL at 26% compared to 50% for bilateral HL. This could be a result of the lower level of suffering with a normal-hearing contralateral ear, but it also could be a result of insufficient provision with HAs.

The grade of HL in persons affected by unilateral HL is mainly in the range of mild to moderate (up to 50 dB HL) according to the WHO grades (Chia et al., 2007). In HA provision, a mild-to-moderate HL often leads to provisions with less occlusion of the ear canal (Kuk and Keenan, 2006; Dillon, 2012). A provision with ear tips is often preferred because of the reduction of the occlusion effect and cost, comfort, and cosmetic factors. Instant ear tips such as open domes lead to lower damping of direct sound reaching the ear canal than closed ear molds (Cubick et al., 2022). Provisions with near-normal transmission of direct sound are often called an “open-fit” (OF) for behind-the-ear HA (Winkler et al., 2016; Cubick et al., 2022). Due to the efficient feedback cancelation and improved wearing comfort, a provision with instant ear tips is often preferred for mild-to-moderate HL (Kiessling et al., 2003; Kuk and Keenan, 2006). OF is the most prominent type of HA fittings nowadays (Cubick et al., 2022).

An OF results in two sound paths, the direct sound and the processed sound. Both reach the eardrum but with a time delay in between and differences in the amplitude spectrum. The processed sound is delayed because of the processing time of the HA (which is abbreviated with the sign *τ* from here on). This means that *τ* superimposes the physiological interaural time difference (ITD) in unilateral HA provision. The ITD results from the difference in path length of a sound arriving at the two ears, and the interaural level difference (ILD) results from the acoustic head shadow at the ear contralateral to the sound source. The ITD in the human auditory system varies between 0 μs for a sound source directly in front of a listener and approximately 700 μs for a sound source at 90° to the side of a listener (Mills, 1958; Thavam and Dietz, 2019). The ITD and the ILD are the two cues to localize sounds in the horizontal plane. They are also important for speech intelligibility in noise when speech and noise sources are not co-located but are spatially separated (Litovsky, 2012; Lavandier and Best, 2020).

Across-frequency delays up to 10 ms have proven to have little or no disturbing effect on speech identification in bilateral HA users (Stone and Moore, 2003). Furthermore, subjective evaluations demonstrated a benefit reported by bilateral HA users for speech intelligibility in noise (Noble and Gatehouse, 2006). For open canal fittings, an acceptable subjective disturbance was reached with delays up to 5 to 6 ms (Stone et al., 2008). These findings were confirmed by Bramsløw (2010) for bilateral HA fittings with a paired comparison task for the preferred settings for sound quality.

To quantify speech intelligibility in noise, the speech reception threshold (SRT) often is reported. The SRT indicates the signal-to-noise ratio (SNR) in dB at which 50% of the speech material is correctly understood (Schädler et al., 2015). Thus, an increase in SRT indicates a loss of speech intelligibility in noise. The SRT of normal-hearing listeners improves when the target speaker and the background noise are spatially separated compared to a situation where they are co-located. This effect is often referred to as spatial release from masking (SRM) and helps listeners understand speech in so-called cocktail party situations (Cherry, 1953; Bronkhorst and Plomp, 1989). SRM highly depends on the correct processing of ITD and ILD in the auditory system (Litovsky, 2012; Glyde et al., 2013). Angermeier et al. (2022) investigated the effect of *τ* on spatial release from masking (SRM) in normal-hearing subjects without direct sound reaching the eardrum(s). Five values of *τ* were imposed on the ITD (0, 1.75, 3.5, 5.25, and 7 ms). To quantify the SRM, the spatial playback configurations with the speech signal from the front at 0° and noise from either 0° (*S*_0°_*N*_0°_) or 90° to the right (*S*_0°_*N*_90°_) were applied. The contributions of ITD and ILD to SRM were studied separately and combined using manipulated head-related impulse responses (HRIR). The procedure resulted in three conditions: (i) only ITD, (ii) only ILD, and (iii) both ITD and ILD. With only ILD, the SRM remained constant, but in the two other conditions, the SRM decreased significantly with increasing *τ*. The decrease in SRM was only dependent on the increase of SRT measured in the spatially separated configuration *S*_0°_*N*_90°_. In the spatially co-located configuration *S*_0°_*N*_0°_, the SRT remained constant over the range of tested *τ*.

Therefore, in the current study, we decided to measure SRTs mainly in the *S*_0°_*N*_90°_ configuration, which is sufficient to study the effect of ITD superposition by *τ* on speech intelligibility in noise. The SRT for normal-hearing listeners is approximately −16 dB when both binaural cues ITD and ILD are present. It increases to −13 dB for ILD only and −12 dB for ITD only (Bronkhorst and Plomp, 1989). However, the study of Angermeier et al. (2022) did not investigate the effect of direct sound that reaches the eardrum in addition to the delayed sound in subjects with unilateral HL and OF. The latency offsets between 1.75 and 10 ms introduced by Angermeier et al. (2022) represent the range of typical values of *τ* of current commercial HA (Stone and Moore, 2003; Bramsløw, 2010; MED-EL Medical Electronics, 2023).

The aim of this study was to investigate the effect of *τ* on speech intelligibility in noise in the presence of simulated OF. The first hypothesis of this study is that direct sound conveys correct ITDs and thus reduces the negative effects of a unilateral *τ* on the SRT a unilateral simulation of OF (further named “uniOF”). The second hypothesis is that different degrees of HL have different effects on the SRT in uniOF. The third hypothesis is that a bilateral simulation of OF (further named “bilOF”) allows binaural processing similar to a situation with two normal-hearing ears. The fourth hypothesis is that the effect of *τ* on SRT in uniOF and bilOF can be predicted with an existing speech intelligibility model. In case the model can replicate our experimental results, it might be a valuable tool to predict the outcome of other values of *τ*, which were not explicitly addressed in this study.

## 2 Materials and methods

### 2.1 Experiment

#### 2.1.1 Subjects

Thirteen normal-hearing subjects (mean age: 25.3 ± 5.8; min: 19; max: 43; 4 women and 9 men) participated in the study. All participants had normal hearing with a hearing threshold at 0.5, 1, 2, and 4 kHz below 20 dB HL (mean thresholds in right ear: 6.0 ± 2.4 dB HL and left ear: 5.6 ± 3.2 dB HL). All participants provided written informed consent. Two participants (male subjects) who participated in the first session could not participate in the second session. The study was conducted following the Code of Ethics of the World Medical Association (Declaration of Helsinki) for experiments involving humans and approved by the Technical University of Munich ethics committee (340/19).

#### 2.1.2 Setup

The experiments were performed in an audiometric booth using the German matrix sentence test “Oldenburger Satztest” (OLSA) (Wagener et al., 1999), with the same experimental setup as used by Angermeier et al. (2022). The stimuli were presented via an external soundcard (RME Fireface 802) and circumaural closed headphones (Sennheiser HD 280 Pro), and participants entered their responses via a tablet (Samsung Tab A) displaying all possible words of the OLSA speech material as a 10 × 5 matrix. A computer outside the booth controlled the experiment. Matlab (The MathWorks Inc. (2021), 9.10.0 (R2021a), Natick, MA, United States) was used to play the stimuli and to receive and analyze the tablet’s responses.

#### 2.1.3 Stimuli

To acquire SRT, the speech material from the OLSA was used with the “Oldenburg noise” (Olnoise) as a masker signal. It consists of stationary noise with a spectrum similar to the long-term spectrum of the speech material. The resulting Olnoise is broadband noise without intelligible speech (Wagener et al., 1999). The noise level was kept constant at 65 dB SPL, and the speech level started at 65 dB SPL (SNR of 0 dB). The speech level was changed adaptively negatively or positively depending on the number of words correctly entered by the participants in each trial. The adaptive change ranged between a step size of 5.848 and 1.462 dB SPL with a conversion target of 0.5 (Wagener et al., 1999).

The speech signal was virtually placed at 0° and the noise signal at 0° or 90° to the right of the listeners. Two different spatial configurations were applied: speech from 0° and noise from 0° (*S*_0°_*N*_0°_) or speech from 0° and noise from 90° (*S*_0°_*N*_90°_). The virtual placement was done by using HRIR. The in-ear HRIRs measured with an artificial head (Bruel & Kjaer 4128C) with the signal presentation at 80 cm distance, 0° elevation, and the azimuth angle of 0° or 90° were used. To do so, in-ear HRIRs from Kayser et al. (2009) were convolved with the speech and noise signals for the left and right ear.

The OF was simulated in two ways.

##### Unilateral OF simulation with direct and delayed sound to the left ear

After HRIR convolution, the speech signal and noise signal for playback in the right ear were added and not further processed to simulate a normal-hearing ear.

The following processing was performed in the same way for speech and noise signals for the left ear.

The convolved signal was duplicated, and the copies of the signals were processed in two different ways. The first path corresponded to the direct sound reaching the left ear without a delay. In an OF with an open dome in the ear canal, the direct sound is almost not attenuated with a small deviation above 2 kHz of approximately 2 dB (Dillon, 2012; Cubick et al., 2022). Thus, it is comparable to an open ear canal.

**Figure 1:**
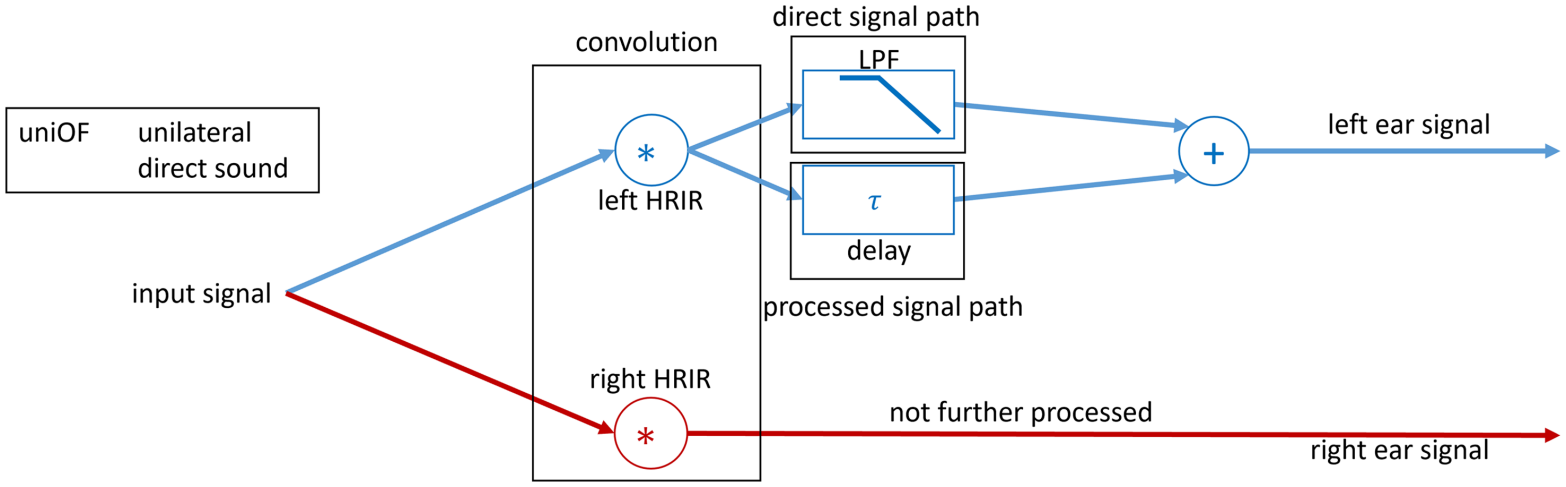
Block diagram of the signal processing used in the study. The spatial separation of the sound sources was realized through convolution of the input speech signal with the 0° HRIR and noise signal with the 90° HRIR. For the left ear, a uniOF is simulated with two signals. The two signals simulate a direct sound with a HL and the delayed sound.

Therefore, the first duplicated signal is attenuated and low-pass filtered (LPF) only to simulate HL (see LPF block in Figure 1). The LPFs are explained in detail in the following subsection (see 2.1.4).

The second copied signal was delayed with the different values of *τ*. For playback on the left ear, both the direct and delayed sounds were then summed (see Figure 1). Angermeier et al. (2022) introduced five values for the latency offset, which corresponds to *τ* in the present study. However, only three of the five values for *τ* were used, namely, 1.75, 3.5, and 7 ms, representing *τ* that are common in commercial HAs (Stone and Moore, 2003; Bramsløw, 2010; MED-EL Medical Electronics, 2023). Finally, the filtered direct sound and the delayed sound were added. Thus, the left ear signal has more energy due to the addition of the two sounds. This reflects the natural conditions for direct and delayed sounds in an OF.

##### Bilateral OF simulation with direct and delayed sound to both ears

**Figure 2:**
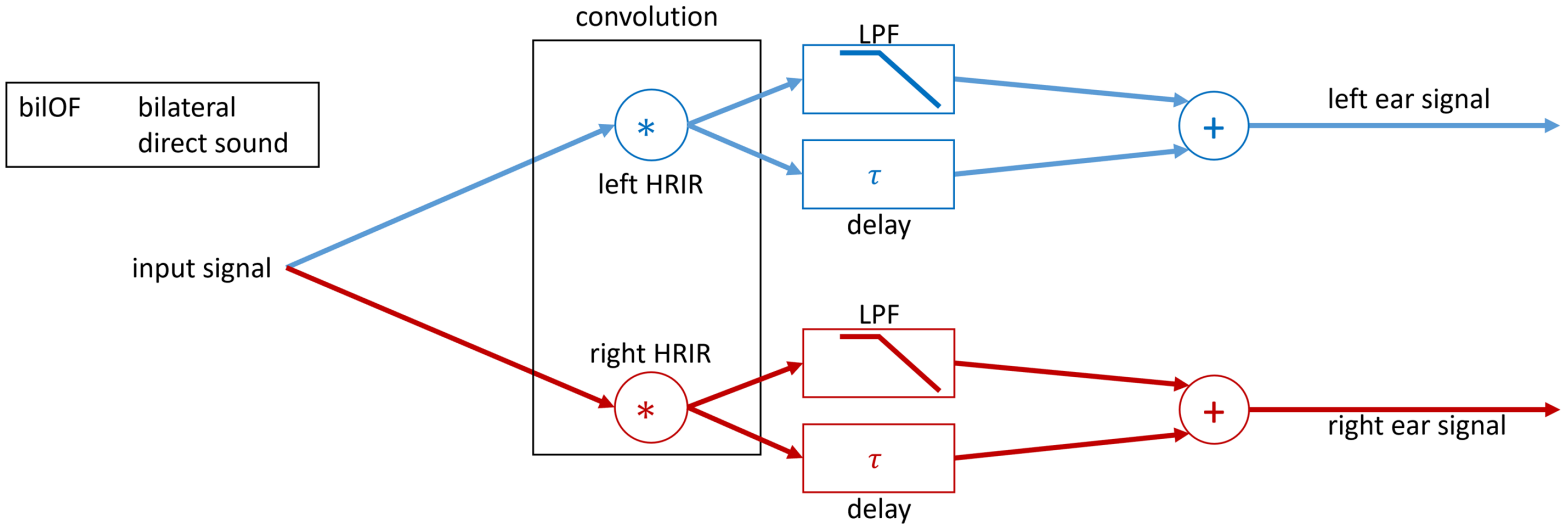
Block diagram of the signal processing used in the study. The spatial separation of the sound sources was realized through convolution of the input speech signal with the 0° HRIR and noise signal with the 90° HRIR. For both ears, a bilOF is simulated with two signals. The two signals simulate a direct sound with HL and the delayed sound.

The bilOF simulation was realized by symmetrical signal processing for both ear signals. The HRIR processing stayed the same as explained in the uniOF section above. The speech and noise signals were processed in the same way. The signals were duplicated on both sides. Then, the two duplicated signals were separately low-pass filtered for the direct sound and delayed for the delayed sound. To get the left and right ear signals for playback, the delayed and direct sounds were summed on both sides (see Figure 2).

##### Reference simulation without direct sound

**Figure 3:**
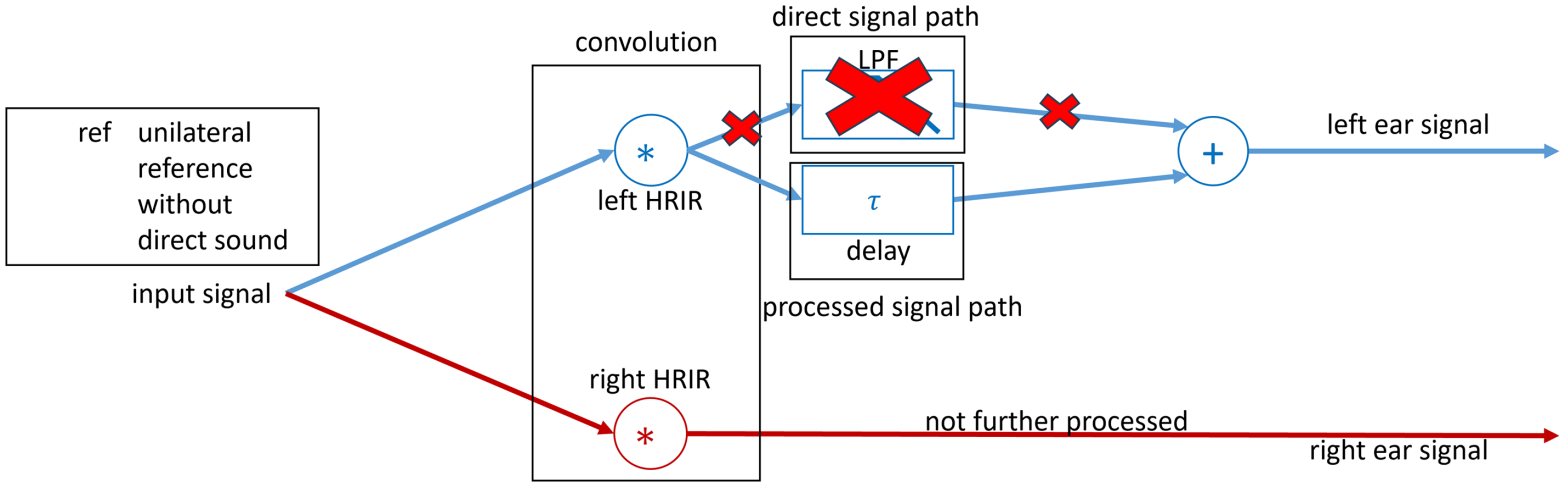
Block diagram of the signal processing used in the study. The spatial separation of the sound sources was realized through convolution of the input speech signal with the 0° HRIR and noise signal with the 90° HRIR. For the left ear, a unilateral closed fit is simulated without direct sound.

To produce a closed fit as a reference to the OF, the direct tone was removed and only the delayed tone was presented (see Figure 3). Thus, the delayed sound was delayed with *τ* = 0 ms and 7 ms for unilateral and bilateral ref. conditions. The *τ* = 0 ms is chosen as a baseline, and *τ* = 7 ms represents the upper limit of common HA delays. The *S*_0°_*N*_0°_ configuration was measured to calculate the initial reference SII for the BSIM2020 model. The initial reference SII is then further used to calculate the SRT of the modeled results.

#### 2.1.4 Hearing loss simulation

**Figure 4:**
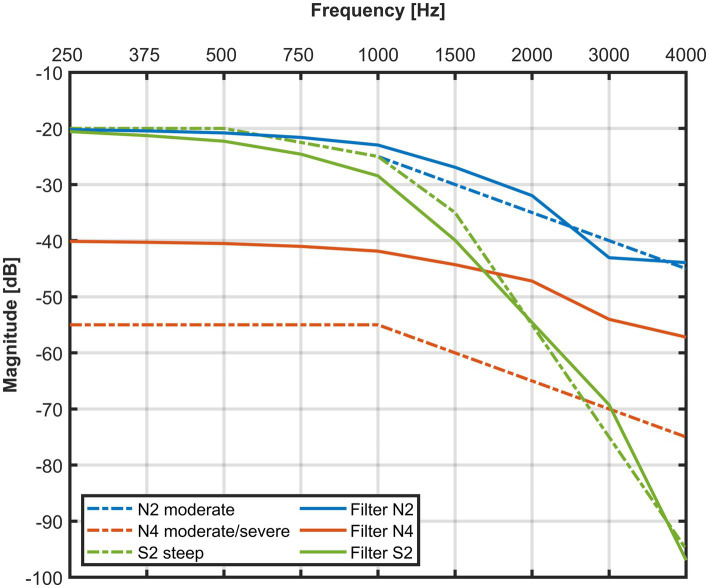
Implemented HL with the dashed lines showing the standard audiograms from Bisgaard et al. (2010), and the solid lines showing the frequency response of the FIR lowpass filters. The 15 dB offset of filter N4 was used as a limiting case before the possible occurrence of feedback and to remain within the range of mild HL according to WHO criteria.

The direct sound was low-pass filtered to simulate the situation in three different HL, which are often referred to as standard HLs (Bisgaard et al., 2010); these are graphically displayed in Figure 4. Two moderately and flat (N2 and N4) and one mild and steep HL (S2) were applied and implemented in two steps. The first step introduced a broadband attenuation of 20 dB (N2 and S2) or 40 dB (N4). The second step consisted of implementing the HL slope with the convolution of the impulse response from a direct-form FIR equiripple zero-phase LPF (see Supplementary Table S1). The LPFs have a constant group delay which was compensated.

#### 2.1.5 Procedure

Testing the two simulations, uniOF and bilOF, was conducted in two separate sessions on different days in random order. At the beginning of the first session, pure-tone thresholds were registered at the frequencies 0.5, 1, 2, and 4 kHz according to the WHO standards to verify normal hearing (Olusanya et al., 2019). Then participants completed two training lists of 20 OLSA sentences in noise (*S*_0°_*N*_0°_ and *S*_0°_*N*_90°_) in each session before starting the actual testing session. The sessions consisted of a total of 12 conditions each, which are listed in Table 1. One OLSA list including 20 sentences per condition was tested.

**Table 1:** Assignment of spatial configuration, LPF types, and *τ* used in the experimental procedure. The first three conditions are reference conditions without a direct sound

Experimental conditions	Spatial configuration	LPF	*τ* (ms)
1	*S*_0°_ *N*_0°_	None	0
2	*S*_0°_ *N*_90°_	None	0
3	*S*_0°_ *N*_90°_	None	7
4	*S*_0°_ *N*_90°_	N2	1.75
5	*S*_0°_ *N*_90°_	N2	3.5
6	*S*_0°_ *N*_90°_	N2	7
7	*S*_0°_ *N*_90°_	N4	1.75
8	*S*_0°_ *N*_90°_	N4	3.5
9	*S*_0°_ *N*_90°_	N4	7
10	*S*_0°_ *N*_90°_	S2	1.75
11	*S*_0°_ *N*_90°_	S2	3.5
12	*S*_0°_ *N*_90°_	S2	7

The sequence of the conditions per session was randomized, and a short recreation break was applied after four and eight lists to reduce fatigue effects. Furthermore, the participants could ask for additional breaks after each list in case of fatigue. Thus, one session with training included a total of 14 lists per participant and lasted approximately 2 h.

The SRT was measured to determine the speech intelligibility of the OLSA speech material in noise under different conditions.

#### 2.1.6 Statistical analysis

Non-parametric statistics were calculated because the data did not have a normal distribution (Shapiro–Wilk *p* < 0.05). The non-parametric Friedmann test with an *α*-level of 0.05 was used to test for the differences between the different LPFs and *τ* combinations and the reference *S*_0°_*N*_90°_ configuration with *τ* = 0 and *τ* = 7 ms. The Friedmann test was used for the simulated uniOF and bilOF separately. If the Friedmann test yielded a significant result, pairwise Wilcoxon signed rank tests with Bonferroni–Holm correction were performed for pairwise comparisons between the reference and the LPF subgroups with *τ* = 7 ms and within the same LPF subgroup with different *τ*.

### 2.2 Model

**Figure 5:**
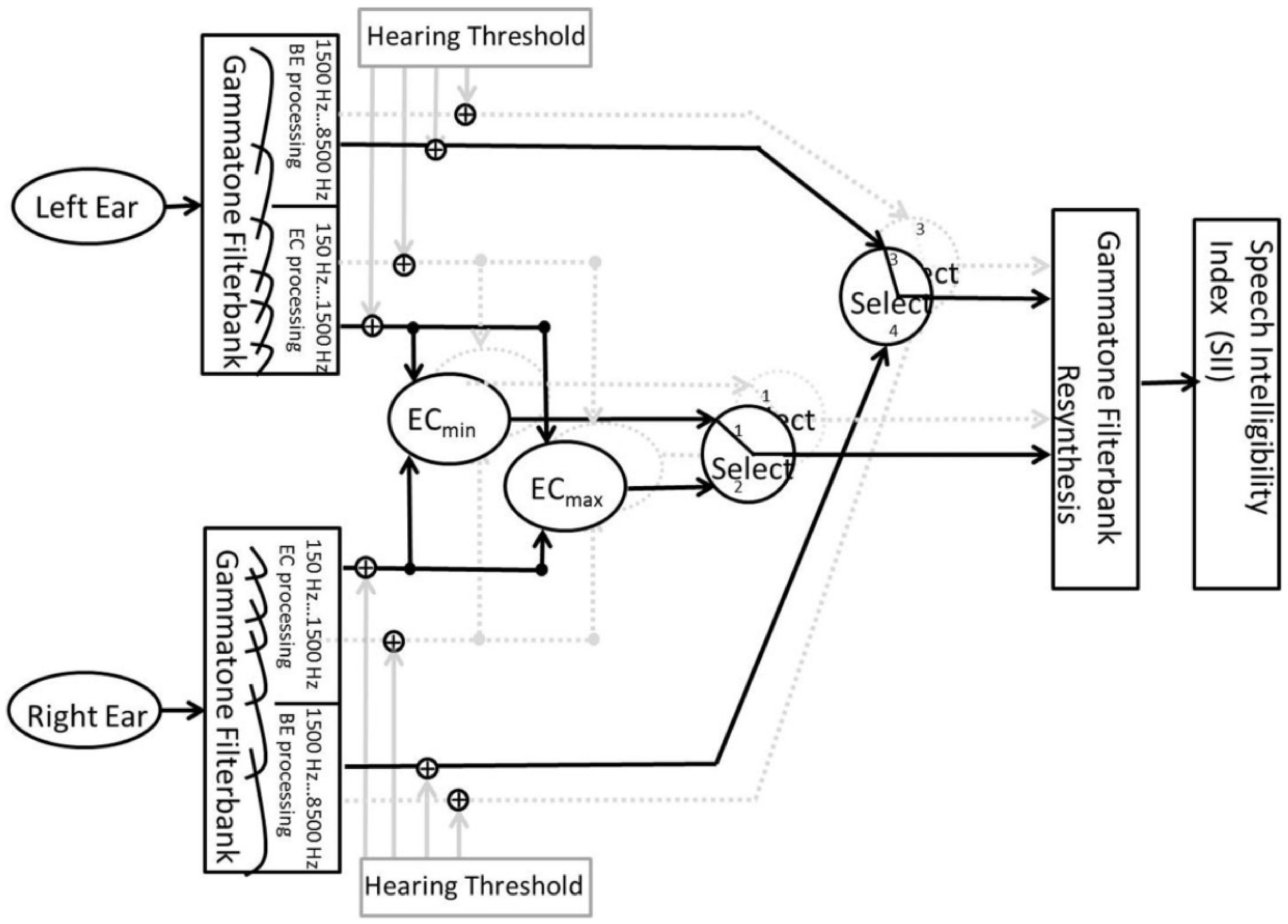
Block diagram of the signal processing of the BSIM2020 model used in this study. The figure is taken from the study by Hauth et al. (2020).

As in the study of Angermeier et al. (2022), the BSIM2020 (Hauth et al., 2020, see Figure 5) implementation from the Auditory Modeling Toolbox was used for the mathematical prediction of SRTs (Majdak et al., 2022).

In the model, the input signals are processed based on better ear listening and binaural unmasking mechanisms. Better ear listening refers to listening with the ear, which is provided with a better SNR. The equalization-cancellation (EC) processing separates the speech from the noise based on the ITD and interaural level difference (ILD). The difference between the left and right ear channels is calculated after ITD and ILD are equalized (Durlach, 1963). These two mechanisms dominate in high frequency (better-ear listening) or low frequency (EC). Therefore, the model splits the input signals from the left and right ear channels into 15 high-frequency (>1,500 Hz) and 15 low-frequency (<1,500 Hz) bands using a gammatone filter bank from 150 Hz to 8,500 Hz (Hohmann, 2002). The high-frequency bands are routed to the better ear selection stage. The low-frequency bands are fed into the equalization-cancellation (EC) processing, which includes two processing paths in the BSIM2020. One path contains the subtraction (EC_min_) and the other contains the addition (EC_max_) of the low-frequency bands from the left and right ear channels, which are equalized for ITD and ILD. Afterward, the two processing paths are forwarded to the EC selection stage. Both selection stages are based on comparing the speech-to-reverberation modulation ratio (SRMR) from Santos et al. (2014) between the two inputs. The SRMR indicates the ratio between the energy of low- and high-modulation frequencies from both inputs. A speech-like modulation is associated with a high SRMR. The EC stage selects between the EC_min_ and EC_max_ processing path, and the better ear stage selects between the left and right ear channels as inputs. Both selection stages’ single outputs are combined into one signal with a gammatone synthesis filter bank. Then, the resynthesized signal is forwarded into the speech intelligibility index (SII) backend (ANSI S3.5-1997, 1997) [see Hauth et al. (2020) for a more detailed model description].

#### 2.2.1 Input signal

The three *τ* values 1.75, 3.5, and 7 ms were used in the experimental procedure and extended with 0, 5.25, and 10 ms, following Angermeier et al. (2022). The three delays were included to verify the entire acceptable delay range specified by Stone and Moore (2003). All six values for *τ*, in combination with the three LPFs, were modeled for both OF simulations. The mixed signals, including speech and noise, were fed as left and right input channels into the BSIM model. Before feeding the signals into the model, the same preprocessing was performed to generate the direct and delayed sounds as in the experiment.

#### 2.2.2 Parameters

An SNR range of 6 to −20 dB in 2 dB steps was used for the speech signal. Ten sentences of the OLSA were modeled for each of these SNRs. To account for the jitter in the EC process, ten Monte-Carlo simulations per sentence were performed for every combination of HL and *τ*. Furthermore, the method from Hauth et al. (2020) was chosen to extract the SRT from the modeled SII data. The mean SII over all Monte-Carlo simulations and sentences from the reference configuration *S*_0°_*N*_0°_ was intersected with the experimentally measured SRT in the same condition. The resulting SII of the *S*_0°_*N*_0°_ configuration was used for all other conditions as reference.

#### 2.2.3 Statistical analysis

A linear regression was performed for the comparison between BSIM and experimental data. MATLAB (Mathworks Inc. (2021), 9.10.0 (R2021a), Natick, MA, United States) was used for statistical testing.

## 3 Results

Since two male participants were unable to take part in the second session, only the results of 11 subjects were analyzed in this section.

### 3.1 Experimental results

**Figure 6:**
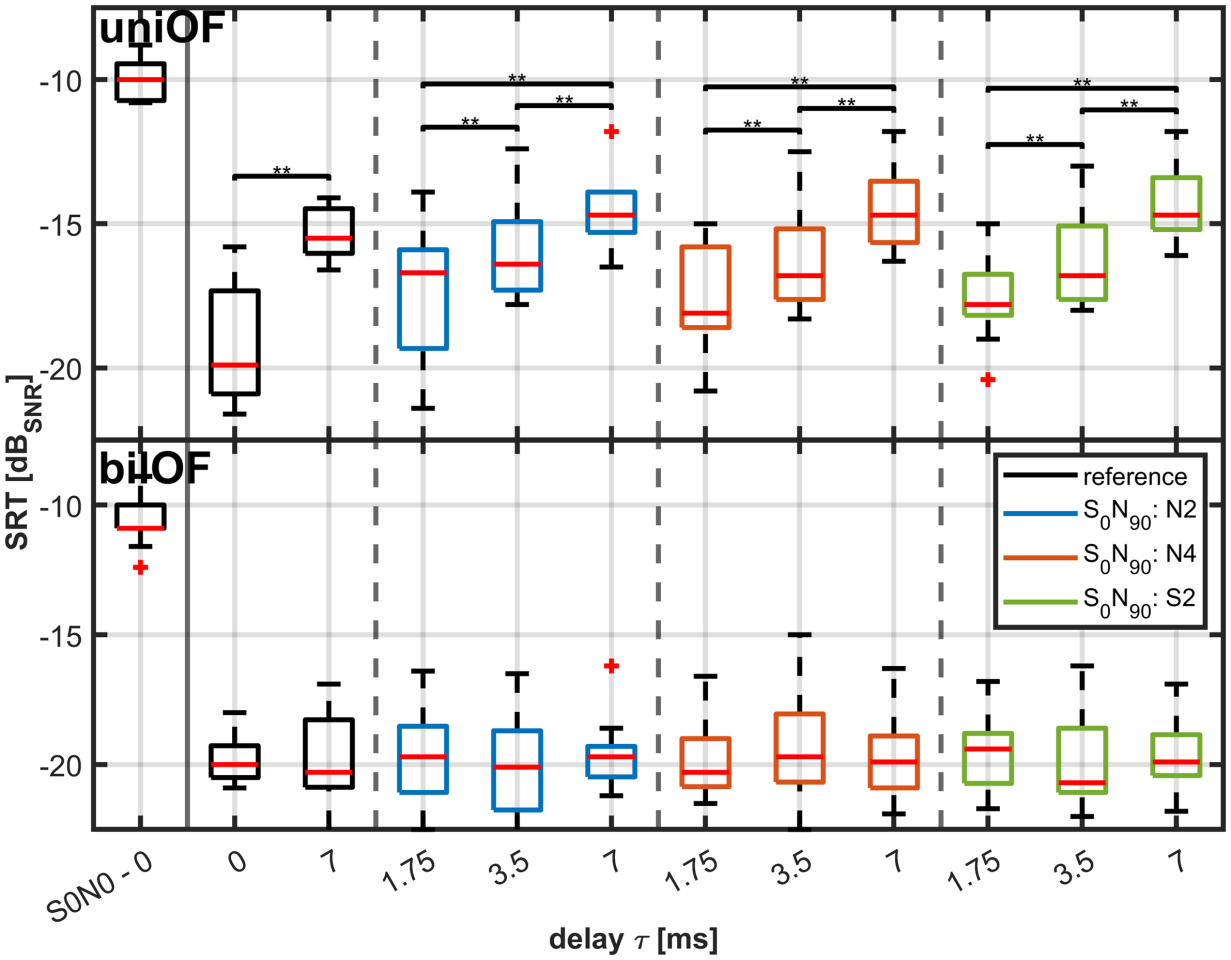
Measured SRTs in 11 normal hearing subjects for *τ* of 0, 1.75, 3.5, and 7 ms as boxplots (red line: median; box: $1^{st}-3^{\mathrm{rd}}$ quartile; whiskers: minimum and maximum without outliers in red). The upper subplot corresponds to the uniOF simulation and the lower subplot to the bilOF simulation. The boxplot on the far left refers to the *S*_0°_*N*_0°_ co-located configuration as a reference for the BSIM2020 model. The other boxplots refer to the SRT in spatially separated configuration *S*_0°_*N*_90°_. The two black boxplots refer to the reference conditions without a direct sound, the blue to LPF N2 condition, the orange to LPF N4 condition, and the green to LPF S2 condition. For better visibility, only the significant pairwise tests are shown with parentheses and asterisks.

Figure 6 shows the measured SRTs as boxplots for the different LPF types and the respective delays *τ*. The black boxplots on the left visualize the results of the reference conditions without a direct sound, i.e., the SRTs in the spatial configuration *S*_0°_*N*_0°_ and *S*_0°_*N*_90°_ with *τ* = 0 and *τ* = 7 ms. The other nine boxplots correspond to the SRTs in the *S*_0°_*N*_90°_ configuration with *τ*. The box colors reflect different LPF types separated by vertical dashed lines. Blue indicates the condition with a direct sound and low-pass filtered with N2; orange is the condition with a direct sound and low-pass filtered with N4; and green is the condition with a direct sound and low-pass filtered with S2. The exact value of the median, 25, and 75% quartile of the data are shown in Table 2.

**Table 2:** Median, 25%, quartile, and 75% quartile of the experimental SRT in uniOF and bilOF simulation across the LPF types and different *τ* in the *S*_0°_*N*_90°_ configuration.

LPF types												
*τ* (ms)	Reference			N2			N4			S2		
	Median	Quartile in %		Median	Quartile in %		Median	Quartile in %		Median	Quartile in %	
		25	75		25	75		25	75		25	75
**Unilateral OF**												
0	−19.9	−20.9	−17.3	—	—	—	—	—	—	—	—	—
1.75	—	—	—	−16.7	−19.3	−15.9	−18.1	−15.8	−18.6	−17.8	−18.2	−16.8
3.5	—	—	—	−16.4	−17.3	−14.9	−16.8	−17.6	−15.2	−16.8	−17.6	−15.1
7	−15.5	−16.0	−14.5	−14.7	−15.3	−13.9	−14.7	−15.7	−13.5	−14.7	−15.2	−13.4
**Bilateral OF**												
0	−20	−20.5	−19.3	—	—	—	—	—	—	—	—	—
1.75	—	—	—	−19.7	−21.1	−18.5	−20.3	−20.9	−19	−19.4	−20.7	−18.8
3.5	—	—	—	−20.1	−21.8	−18.7	−19.7	−20.7	−18.1	−20.7	−21.1	−18.6
7	−20.3	−20.9	−18.3	−19.7	−20.5	−19.3	−19.9	−20.9	−18.9	−19.9	−20.4	−18.9

For analysis, the Friedman test was applied to the data of the different LPF types and the reference with a delay of *τ* = 0 ms and 7 ms. The Friedman test revealed a significant effect (*χ*^2^ (10) = 86.93, *p* < 0.01).

Pairwise tests were performed for the comparisons of the uniOF simulation conditions. The comparisons were split into two groups to analyze the effect of both parameters, the delay *τ* and the different LPF types, separately. Thus, a total of 25 pairwise tests were performed. The tests were divided into two groups: 10 for the variation of delay and 15 for the different HL simulations (see Supplementary Table S2).

In both groups, the Bonferroni–Holm correction was applied.

For *τ*, a significant difference was found between the two reference conditions without a direct sound and with *τ* = 0 ms and *τ* = 7 ms (*p* = 0.008) and within the different LPF types with direct sound but different values of *τ*. The pairwise comparisons revealed significant differences between the three different delays within all three LPF types (*p* < 0.05, see Supplementary Table S3).

In the second group, the LPF types with the same values of *τ* were compared. No significant difference in the pairwise comparisons between the different LPF types with the same *τ* was revealed. Therefore, LPF showed no effect on SRT, but *τ* showed an increasing effect on SRT.

For bilOF simulation, the median SRT across the LPF types and the reference with *τ* = 0 ms and *τ* = 7 ms revealed no significant effect of *τ* on SRT (*χ*^2^ (3) = 1.89, *p* = 0.303). No pairwise tests were applied to the bilOF simulation conditions due to the lack of effect shown in bilOF simulation by the Friedman tests.

A significant difference was also revealed by the Friedman test applied for the median SRT of uniOF and bilOF (*χ*^2^ (23) = 205.55, *p* < 0.01). The pairwise testing was performed between the results in the uniOF simulation compared to the corresponding results in the bilOF simulation. All pairwise comparisons between uniOF and bilOF yielded significant differences except for the two reference conditions without a delay (*p* < 0.05, see Figure 6 and Supplementary Table S4).

In conclusion, there was no significant effect of the direct sound across the LPF types on SRT shown. The simulated direct sound did not influence the SRT.

In contrast, a significant effect of *τ* on SRT was present in the uniOF simulation but not in the bilOF simulation. In the uniOF simulation, the SRT increased with increasing *τ*. This effect was present across all LPF types.

### 3.2 Modeling results

**Figure 7:**
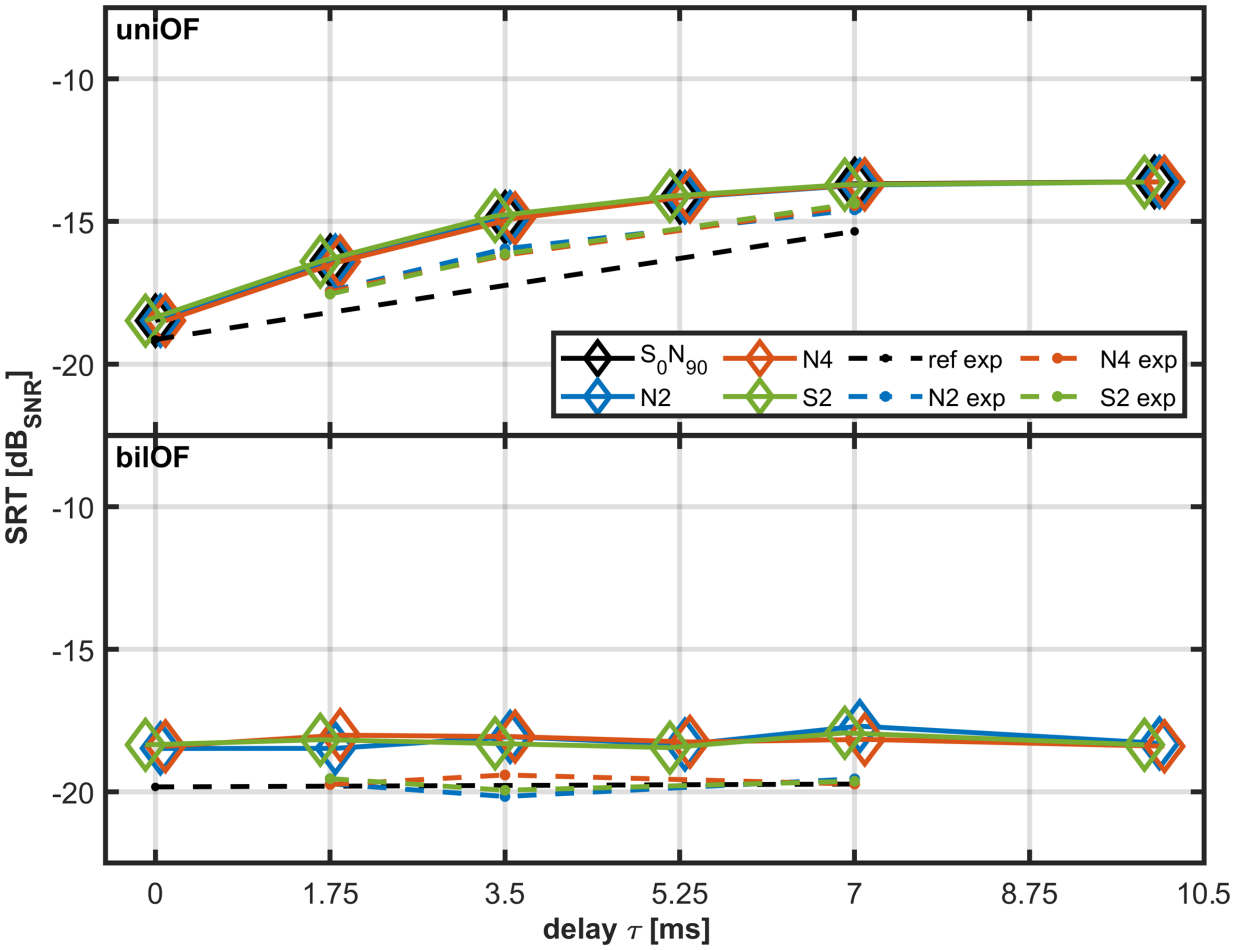
Modeled SRTs for *τ* of 0, 1.75, 3.5, 5.25, 7, and 10 ms for *S*_0°_*N*_90°_ as diamonds. The upper subplot corresponds to the uniOF simulation and the lower subplot to the bilOF simulation. The reference conditions (ref) are displayed in black, the LPF N2 conditions in blue, the LPF N4 conditions in orange, and the LPF S2 conditions in green. The experimental data with the different LPFs are shown with dashed lines (N2 exp., N4 exp., and S2 exp), with the same colors for the different LPFs. The different markers were jittered for better visibility.

Figure 7 depicts the modeled SRTs for *S*_0°_*N*_90°_ as diamonds. The upper plot shows the modeled SRTs of the uniOF simulation, and the lower plot shows the modeled SRTs of the bilOF simulation. The model results for the uniOF simulation show a comparable influence of *τ* on the SRT as the experimental results with the uniOF simulation. Similar to the experimental results, the modeled SRTs within the uniOF simulation increase with increasing *τ* (up to 10 ms) in the *S*_0°_*N*_90°_ configuration. This is reflected in a high *R*^2^ value and a low RMSE for the uniOF simulation. The variance of the SRT is mainly explained by the change in *τ*.

In contrast, the variation of the bilOF simulation SRT cannot be explained by the increase in *τ*, reflected by the low-adjusted *R*^2^ value. The variation of the SRT can mainly be explained by increasing *τ* for the uniOF simulation, which is shown by the high-adjusted *R*^2^ values (Table 3 upper part). Calculating *R*^2^ in the modeled *S*_0°_*N*_90°_ configuration within bilOF simulation yielded low values (see Table 3 lower part), consistent with the flat line in the lower plot of Figure 7 for increasing *τ*.

**Table 3:** Coefficients of determination (*R*^2^) and root-mean-square errors (RMSE) for the linear regression between modeled results for uniOF and bilOF simulation for different *τ* with the same LPF type.

LPF type	N2			N4			S2		
	*R* ^2^	RMSE (dB)	*p*-Value	*R* ^2^	RMSE (dB)	*p*-Value	*R* ^2^	RMSE (dB)	*p*-Value
**Unilateral OF simulation (uniOF)**									
SRT	0.80	0.95	0.02	0.80	0.95	0.02	0.79	0.97	0.02
**Bilateral OF simulation (bilOF)**									
SRT	0.22	0.30	0.35	0.07	0.18	0.61	0.01	0.20	0.83

## 4 Discussion

In situations where the target speaker and background noise are spatially separated, binaural processing allows for better speech intelligibility of the target speaker compared to a situation where the target speaker and background noise are co-located. In the spatially separated condition, the SRT depends strongly on the correct processing of the ITD and ILD in the auditory system (Litovsky, 2012; Glyde et al., 2013). A unilateral delay *τ*, e.g., introduced by the processing of an HA, hampers speech intelligibility in noise, as shown by Angermeier et al. (2022). More precisely, the SRT in the spatially separated *S*_0°_*N*_90°_ configuration deteriorates with increasing *τ* (0…10 ms). Those values are typical for processing delays in current digital HAs (Stone and Moore, 2003; Bramsløw, 2010; MED-EL Medical Electronics, 2023). One phenomenon in recent HA fittings is that due to efficient feedback cancellation and improved wearing comfort, OF with instant ear tips are very commonly used nowadays instead of closed earmolds (Cubick et al., 2022). In such cases, not only the amplified sound from the HA but also direct sound reaches the ear drum.

Our first hypothesis of the study was that the presence of direct sound, which conveys correct ITDs, reduces the negative effects of a unilateral *τ* on the SRT. In the uniOF simulation, direct and delayed sounds were present in one ear, and the other ear signal was unprocessed. To generate a realistic type of direct sound, it was processed with different LPFs mimicking three different HLs. The simulated HL corresponded to the most common grades of mild and moderate HL (Bisgaard et al., 2010). The results in the uniOF simulation showed a significant increase (i.e., deterioration) of SRT in the *S*_0°_*N*_90°_ configuration with increasing *τ*. A severe deterioration is evident at 7 ms, where the limit of binaural deterioration is reached and only speech intelligibility with the ear with the better SNR due to the head shadow effect remains. We substantiate this statement by referencing to Angermeier et al. (2022), who showed that when only ILD are available to listeners the SRM is as large as 4.8 dB on average. This remaining SRM, which is much smaller than when both ITD and ILD are available to listeners (8.8 dB on average), is not due to binaural processing but only to “better ear listening” (Litovsky, 2012).

Direct sound did not help to prevent the deterioration of SRTs with increasing *τ* in the uniOF simulation. This result is consistent with the concept of backward masking from a delayed sound source described by Blauert (1997). Backward masking of the direct sound occurs when the level of the direct sound signal is 10 dB or lower than the delayed signal (Dillon, 2012). Our results confirm this at a level difference of more than 20 dB in our HL conditions. As a result, the delayed signal dominates the perception, and therefore the direct sound, in our case, is negligible for the perception.

Due to the backward masking of the direct sound, an effect of *τ* on SRT comparable to Angermeier et al. (2022) is observed. This finding suggests that asymmetrical signal processing with direct and delayed sounds from the HA affects the binaural processing of spatially separated speech and noise.

The second hypothesis was that different degrees of HL have different effects on the SRT with uniOF simulation. The results showed no significant difference in SRT in the *S*_0°_*N*_90°_ configuration with different LPF types for the same *τ*, respectively. To conclude, the different simulated HL types did not affect binaural processing because of the previously mentioned backward masking.

The third hypothesis was that bilOF simulation reveals binaural processing similar to a situation with two normal-hearing ears. In the corresponding bilOF simulation, the direct and delayed sounds are present in both ears. The direct sound was again generated with the same LPF as in the uniOF simulation mimicking three different HL (Bisgaard et al., 2010). In the bilOF simulation, in contrast to the finding with the uniOF simulation, no significant effect of increasing *τ* on SRT was observed. This finding suggests that symmetrical signal processing with direct and delayed sounds from the HA does not affect binaural processing of speech and noise. The results in the bilOF simulation are consistent with the findings of McArdle et al. (2012) and Dawes et al. (2013), who also reported better speech intelligibility for bilateral provision in comparison to unilateral provision in HA users with symmetric HL due to preservation of ITD and ILD.

The fourth hypothesis was that the effect of *τ* on SRT can be predicted with an existing speech intelligibility model. The results showed that the accuracy of the SRTs predicted with the applied BSIM2020 model was high. The negligible influence of the direct sound on the experimental data was also evident for the level equalization in the equalization-cancellation process in the BSIM model as the level difference between direct and delayed signal was larger than 10 dB across all frequency bands. Thus, the delayed signal dominates the time-dependent equalization-cancellation processing. In the model, the influence of *τ* on SRT can be explained by the proportional increase of processing errors within the time-sensitive EC process (Vom Hövel, 1984).

The BSIM model predicted a constant SRT even with increasing *τ* for the bilOF simulation. This is well in line with the experimental outcomes of this study and complements the results from Bramsløw (2010), who showed no significant effect of a HA delay from 5 up to 10 ms on modeled SII. The BSIM model seems to be a tool of choice for predicting SRT for standard HL in the uniOF and bilOF simulation. Furthermore, the model can be used to predict the impact of the processing time of novel signal processing strategies on SRT. Such novel signal processing strategies for modern HA should enable listeners to make use of binaural cues and not just better ear listening.

In conclusion, delays of up to 10 ms, as those mentioned in Stone and Moore (2003), Bramsløw (2010) and by MED-EL Medical Electronics (2023), are suitable for sufficiently good speech identification in bilateral HA users. Our results suggest that for improved SRT in unilateral HA users, smaller processing delays are needed. Smaller processing delays in digital signal processing are possible, for example, by reducing the frame size or by increasing the sampling rate. Reducing the frame size also results in a loss of spectral resolution. However, for unilateral HL, a reduced number of frequency bands with adjustable amplification and compression may not be a major disadvantage for HA fitting because the common mild-to-moderate high-frequency HL with near-normal hearing thresholds in the low frequencies does not necessarily require amplification in the low frequencies. Therefore, rapid signal processing algorithms with a limited amount of frequency bands with adjustable amplification and compression in the mid and high frequencies might be a reasonable approach for unilateral HA provision in many cases. A higher sampling rate also causes higher power consumption and therefore reduced battery lifetime. Thus, a trade-off must be made between reducing the processing delay and tolerable battery runtime.

### 4.1 Limitations of this study

No actual HA users were tested. Furthermore, only an ideal HA simulation recovering full audibility was simulated in both the experiment and the model. HL was simulated by an attenuative filter, i.e., simulating conductive HL. Recruitment, a common feature of sensorineural HL, was not simulated. Furthermore, the static positioning of the noise source (at 90° azimuth) and speaker (at 0° azimuth) is somewhat artificial. Moreover, room reflections were not simulated which could help to increase realism. However, the results are still valid for a relative comparison of SRTs in unilateral and bilateral HA simulations with/without direct sound.

## 5 Conclusion

The outcomes of our study revealed that the simulation of relatively small processing delays in the range of 3 to 10 ms, as they occur in current commercial HAs, hamper speech intelligibility in noise. This effect was observed in normal-hearing listeners using a simulated unilateral HA with OF. The reduction of speech intelligibility was evident when speech and noise sources were spatially separated as is often the case in real-world listening scenarios. The direct sound of the simulated OF did not provide any benefit for speech intelligibility in noise. In contrast, no significant effect of a simulated bilateral HA with OF on SRT was found. The outcomes emphasize the development of rapid signal processing algorithms for unilateral HA provision with OF.

## Data availability statement

The raw data supporting the conclusions of this article will be made available by the authors, without undue reservation.

## Ethics statement

The studies involving humans were approved by Ethics Committee of the Technical University of Munich. The studies were conducted in accordance with the local legislation and institutional requirements. The participants provided their written informed consent to participate in this study.

## Author contributions

SR: conception and design, acquisition and interpretation of data, and final approval. F-UM and JA: conception and revision. WH and SZ: supervision, conception, revision, and final approval. All authors contributed to the article and approved the submitted version.
