# Supplementary material for: Effect of a processing delay between direct and delayed sound in simulated open fit hearing aids on speech intelligibility in noise
Source: Front Neurosci. 2024 Jan 4;17:1257720. doi: 10.3389/fnins.2023.1257720 (PMC10805375; doi:10.3389/fnins.2023.1257720)
Supplement: Supplementary file 1 [file Data_Sheet_1.pdf]

Supplementary material

Table 1: FIR low-pass filter parameters for the HL simulations.

LPF type	color/ line style	order	passband	stopband
N2	blue/ solid	45	0.5 kHz	6 kHz
N4	orange/ solid	38	1 kHz	4 kHz
S2	green/ solid	89	0.5 kHz	6 kHz

Table 2: The pairwise comparisons for the two parameters.

**LPF**	**τ**	**vs**	**LPF**	**τ**
		**Varied LPF**		
ref	7	vs	N2	7
ref	7	vs	N4	7
ref	7	vs	S2	7
N2	1.75	vs	N4	1.75
N2	1.75	vs	S2	1.75
N4	1.75	vs	S2	1.75
N2	3.5	vs	N4	3.5
N2	3.5	vs	S2	3.5
N4	3.5	vs	S2	3.5
N2	7	vs	N4	7
N2	7	vs	S2	7
N4	7	vs	S2	7
N2	1.75	vs	N4	1.75
N2	1.75	vs	S2	1.75
N4	1.75	vs	S2	1.75
		**Varied τ**		
ref	0	vs	ref	7
N2	1.75	vs	N2	3.5
N2	1.75	vs	N2	7
N2	3.5	vs	N2	7
N4	1.75	vs	N4	3.5
N4	1.75	vs	N4	7
N4	3.5	vs	N4	7
S2	1.75	vs	S2	3.5
S2	1.75	vs	S2	7
S2	3.5	vs	S2	7

Table 3: p-values from the Wilcoxon signed rank pairwise comparisons within LPF types in the uniOF simulation.

vs	ref	N2			N4			S2		
	0	1.75	3.5	7	1.75	3.5	7	1.75	3.5	7
1.75	-	-	0.03	0.01	-	0.02	0.01	-	0.01	0.01
3.5	-	-	-	0.01	-	-	0.01	-	-	0.01
7	0.01	-	-	-	-	-	-	-	-	-

Table 4: p-values from the Wilcoxon signed rank pairwise comparisons between corresponding LPF types of bilOF and uniOF simulation.

	Reference			N2			N4			S2		
	$S_{0^\circ}N_{0^\circ}$	0	7	1.75	3.5	7	1.75	3.5	7	1.75	3.5	7
bilOF vs uniOF	0.13	0.38	0.01	0.02	0.01	0.01	0.02	0.01	0.01	0.02	0.01	0.01
